# Role of Maternal Periodontitis in Preterm Birth

**DOI:** 10.3389/fimmu.2017.00139

**Published:** 2017-02-13

**Authors:** Hongyu Ren, Minquan Du

**Affiliations:** ^1^MOST KLOS and KLOBM, School and Hospital of Stomatology, Wuhan University, Wuhan, China

**Keywords:** periodontitis, pregnancy, preterm birth, low birth weight, risk factor

## Abstract

In the last two decades, many studies have focused on whether periodontitis is a risk factor for preterm birth (PTB). However, both epidemiological investigation and intervention trials have reached contradictory results from different studies. What explains the different findings, and how should future studies be conducted to better assess this risk factor? This article reviews recent epidemiological, animal, and *in vitro* studies as well as intervention trials that evaluate the link between periodontitis and PTB. Periodontitis may act as a distant reservoir of microbes and inflammatory mediators and contribute to the induction of PTB. Animal studies revealed that maternal infections with periodontal pathogens increase levels of circulating IL-1β, IL-6, IL-8, IL-17, and TNF-α and induce PTB. *In vitro* models showed that periodontal pathogens/byproducts induce COX-2, IL-8, IFN-γ, and TNF-α secretion and/or apoptosis in placental tissues/cells. The effectiveness of periodontal treatment to prevent PTB is influenced by the diagnostic criteria of periodontitis, microbial community composition, severity of periodontitis, treatment strategy, treatment efficiency, and the period of treatment during pregnancy. Although intervention trials reported contradictory results, oral health maintenance is an important part of preventive care that is both effective and safe throughout pregnancy and should be supported before and during pregnancy. As contradictory epidemiological and intervention studies continue to be published, two new ideas are proposed here: (1) severe and/or generalized periodontitis promotes PTB and (2) periodontitis only promotes PTB for pregnant women who are young or HIV-infected or have preeclampsia, pre-pregnancy obesity, or susceptible genotypes.

## Introduction

Each year, about 15 million infants worldwide are born preterm (before 37 weeks of gestation), and these preterm babies typically have low birth weight (LBW, <2,500 g) (1). Preterm birth (PTB) is the leading cause of neonatal mortality, morbidity, and developmental loss (2). Advances in obstetric care have not altered the rates of PTB, and it is estimated that 9.6% of worldwide births are preterm (3). The highest rates of PTB are in Africa (11.9%) and North America (10.6%), and the lowest rates are in Europe (6.2%) (3). However, the underlying causes of PTB are still not entirely clear, thus an accurate identification of risk factors for PTB that are amenable to intervention would have far-reaching and long-lasting impact.

**Figure 1:**
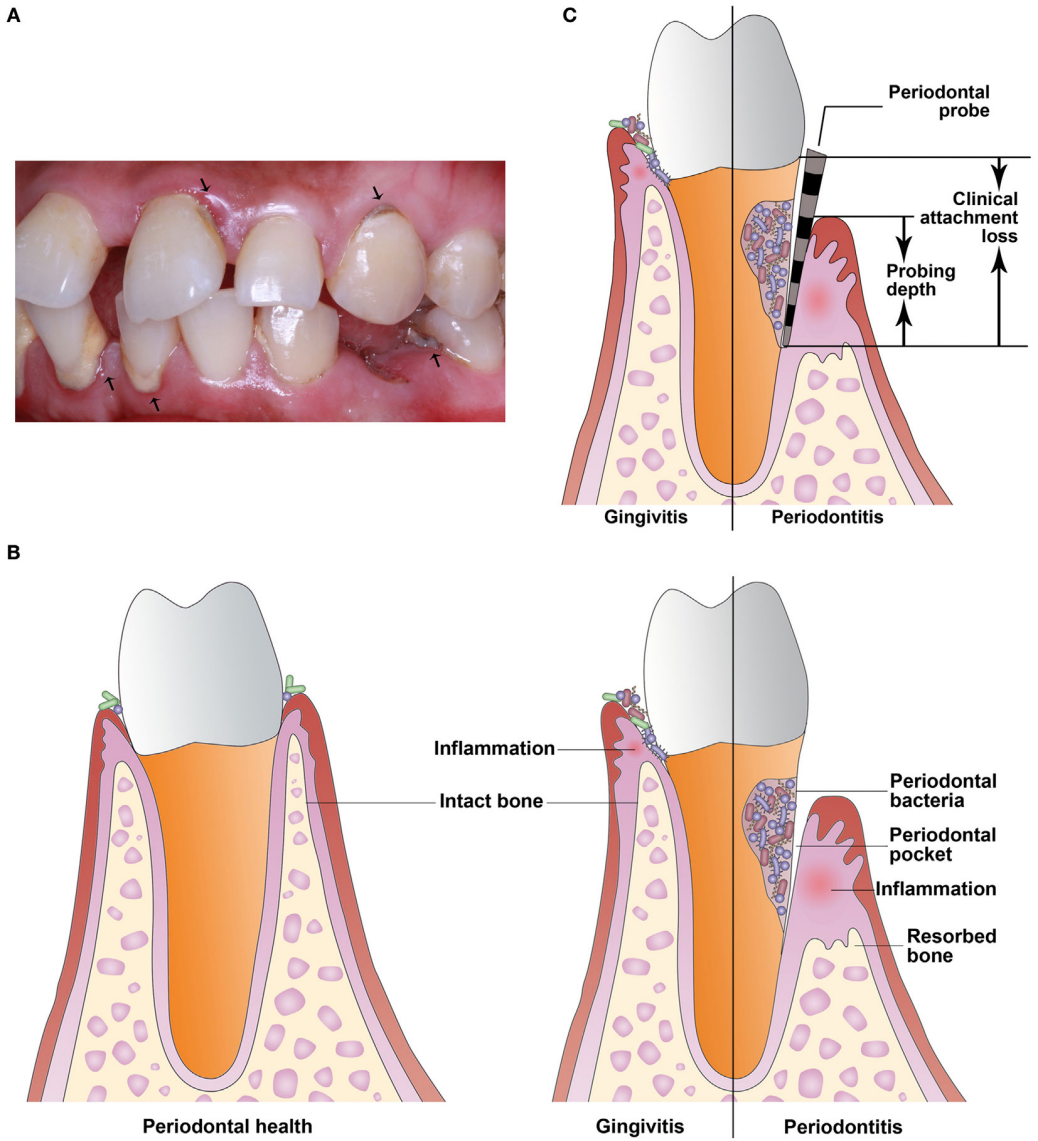
**Periodontal disease is a highly prevalent infectious and inflammatory disease of tooth-supporting tissues**. **(A)** The arrowheads indicate periodontal disease. **(B)** Periodontal disease includes gingivitis and periodontitis. Gingivitis is the presence of gingival inflammation without loss of connective tissue attachment. Periodontitis is the presence of gingival inflammation at sites where there has been apical migration of the epithelial attachment onto the root surfaces accompanied by the loss of connective tissue and alveolar bone. **(C)** Clinical attachment loss is measured with a periodontal probe and is the distance from the base of the probeable crevice to the cementoenamel junction. Probing depth is defined as the distance between the bottom of the periodontal pocket and the gingival margin.

Of the multiple risk factors for PTB, maternal infection is identified consistently. Periodontal disease is a highly prevalent infectious and inflammatory disease of tooth-supporting tissues and if untreated can lead to oral disabilities (Figure 1A) (4). Periodontal disease is caused mainly by gram-negative microaerophilic and anaerobic bacteria that colonize the subgingival area and produce significant amounts of proinflammatory mediators (5). Periodontal disease includes gingivitis and periodontitis. Gingivitis is the presence of gingival inflammation without loss of connective tissue attachment. Periodontitis is the presence of gingival inflammation at sites where there has been apical migration of the epithelial attachment onto the root surfaces accompanied by the loss of connective tissue and alveolar bone (Figure 1B) (6). In the last two decades, many studies have examined the relationship between periodontitis and PTB. Periodontitis may be a risk factor for PTB due to the presence in the bloodstream of bacteria and proinflammatory cytokines during infection that can affect distant organs (7). However, epidemiological studies and intervention trials have reached contradictory conclusions about the relationship between periodontitis and PTB. The aim of this review is to summarize the current evidence from epidemiological, animal, and *in vitro* studies, as well as intervention trials and to propose new ideas about the link between periodontitis and PTB.

## Epidemiological Results

About one-third of all PTB are caused by preterm labor (uterine contraction) and one-third are due to the premature rupture of membranes (PROM); the remaining cases are due to other pregnancy complications such as induced labor (of which preeclampsia is the major indication) (8). In the last two decades, numerous epidemiological studies have examined the link between periodontitis and PTB, including cross-sectional, case–control, and cohort studies.

In a cross-sectional study, or census, data are collected at a defined time and is used to assess the prevalence of chronic or acute conditions, the results of intervention, or the causes of disease. In the last 5 years, several cross-sectional studies (9–11) reported a correlation between periodontitis and PTB/LBW. In a study published in 2016 (9), women with PTB were found to have worse periodontal parameters and significantly increased gingival crevicular fluid (GCF) levels of IL-6 and prostaglandin E_2_ (PGE_2_) compared with women who experienced full-term birth. Based on significant correlations between serum PGE_2_ level and probing depth, clinical attachment loss (CAL, Figure 1C), and GCF TNF-α in PTB, periodontitis may increase the risk of labor triggers and hence contribute to preterm labor onset. However, in 2016 Martinez-Martinez et al. (12) suggested that PTB is a multifactorial condition and that periodontitis and the presence of periodontal pathogens are not sufficient to trigger PTB.

In case–control studies, mothers with PTB are identified and their periodontitis history is determined and compared with that of healthy control subjects. Of the 14 case–control studies published in the last 5 years, 12 (13–24) reported an association between periodontitis and PTB, LBW, or preterm LBW (PLBW), and 2 (25, 26) found no association. In a study published in 2015 (23), mothers in the periodontitis group with single delivery had an eightfold higher chance of delivering a LBW infant compared to those in the control group. The mothers in the periodontitis group with multiple deliveries delivered PTB infants with an eightfold higher frequency and LBW infants at a 10-fold higher frequency compared to the mothers in the control group.

In cohort studies, investigators monitor pregnant women to determine if those with periodontitis demonstrate a higher incidence of PTB than those without periodontitis. Of the 11 published cohort studies in the last 5 years, 7 (27–33) reported an association between periodontitis and PTB, LBW, or PLBW, and 4 (34–37) revealed no association. A hospital-based prospective study published in 2016 (33) comprising 790 pregnant women found that periodontitis was a risk factor for PTB and an independent risk factor for LBW. Recently periodontitis was also found to be associated with preeclampsia (38) and PROM (39, 40), common causes of PTB.

In the last 5 years, most literature and systematic reviews reported an association between periodontitis and PTB (41–46). A meta-analysis published in 2016 (41) assessed case–control studies reporting periodontal status and pregnancy outcomes. The computed risk ratio for periodontitis was 1.61 for PTB using data from 16 studies, the risk for LBW was 1.65 based on 10 studies, and the risk for PLBW was 3.44 based on 4 studies. A systematic and evidence-based review in 2012 (46) focused on the association of periodontitis and PTB and LBW and found 62 relevant studies that suggested that periodontitis may be a potential risk factor for PTB and LBW.

Different epidemiological data may have reached different conclusions due to the following reasons: (1) many studies used different definitions of periodontitis and adverse pregnancy outcomes, for instance the use of PLBW as a composite outcome, or PTB versus LBW, terms that reflect different disease severities and pathologic entities. (2) The risk factors of PTB may be similar to the risk factors for periodontitis (ethnicity, tobacco use, and socioeconomic and educational levels) and thus may confound the association between periodontitis and PTB. In a prospective case–control study (47), PTB was associated with periodontitis when the USA (48), but not the European (49), definitions were used. Therefore, future studies should employ both continuous and categorical assessment of periodontal status and control for confounding factors. Additionally, the further use of the composite outcome PLBW is not encouraged.

## Biological Hypotheses

**Figure 2:**
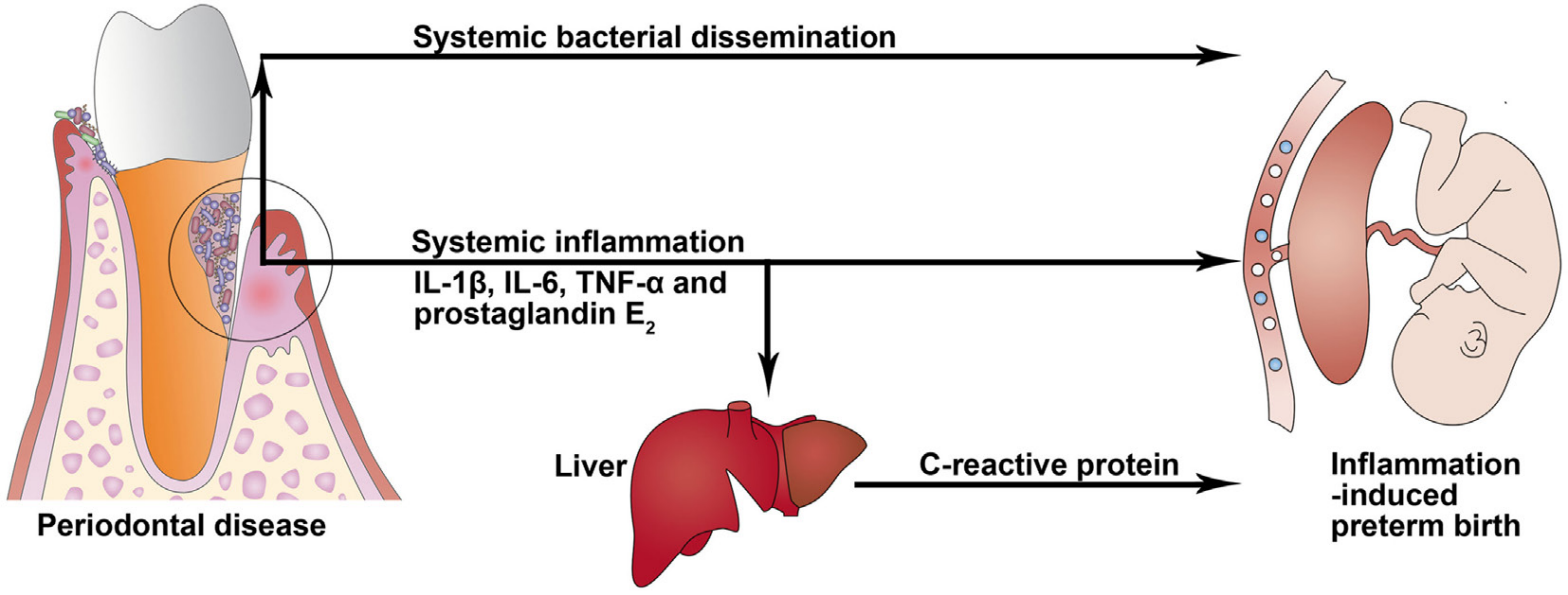
**Potential biological mechanisms linking periodontal disease to preterm birth**. In periodontitis, gingival ulceration in the periodontal pocket enables egress and systemic bacterial dissemination, and locally produced proinflammatory cytokines can enter systemic circulation and induce an acute-phase response in the liver that is characterized by an increased level of C-reactive protein.

Periodontitis is one of the most common chronic infectious diseases and is caused mainly by gram-negative microaerophilic and anaerobic bacteria that colonize the subgingival area and produce significant amounts of proinflammatory mediators, mainly IL-1β, IL-6, PGE_2_, and TNF-α (4). Periodontitis may act as a distant reservoir of both microbes and inflammatory mediators that may influence pregnancy and contribute to induction of PTB (Figure 2). These two potential mechanisms for how periodontitis can affect PTB are described more fully below.

### Bacterial Spreading

Periodontal microorganisms can act as pathogens not only in the oral cavity but also in other body areas. This is due to the following characteristics of bacteria: (1) the ability to rapidly colonize, (2) the ability to elude the host’s defense mechanisms, and (3) the ability to produce substances that directly contribute to the destruction of tissue. Periodontal pathogens/byproducts may reach the placenta and enter the amniotic fluid and fetal circulation, serving to activate inflammatory signaling pathways.

*Porphyromonas gingivalis* has been detected in human placenta tissues (50). Interestingly, one study reported that *P. gingivalis* was only detected within the villous mesenchyme in the preterm cohort, but not the term group (51). Thus, the detection of *P. gingivalis* in the placenta may be related to PTB (52, 53).

The *Fusobacterium nucleatum* subsp. *polymorphum* strain was not detected in vaginal samples, but was found in both neonatal gastric aspirates and oral samples from mothers with PTB and localized periodontal pockets, which strongly indicated that *F. nucleatum* subsp. *polymorphum* of oral origin may relate to PTB (54). Bohrer et al. (55) reported a case of acute chorioamnionitis caused by *F. nucleatum* that progressed to maternal sepsis in a term patient with intact membranes. Cassini et al. (56) reported that the presence of periodontal pathogen *Treponema denticola* in the vagina, regardless of the levels, increased risk of PTB.

Levels of *P. gingivalis, F. nucleatum, Actinomyces actinomycetemcomitans, Tannerella forsythia, T. denticola, Eikenella corrodens*, and *Capnocytophaga* spp. have been reported at significantly higher levels in preterm deliveries as compared to term births (37, 52, 57). In one study (58), when *Prevotella intermedia* and/or *Aggregatibacter actinomycetemcomitans* were not detected in maternal periodontal pockets, the infants were more than 129% likely to have a normal birth weight.

The above findings suggest that periodontal bacteria may be normally present in the placenta. However, the levels of certain periodontal pathogens in the placenta may be dependent on the maternal periodontal state (59). Further studies are needed to elucidate the role of microbial load and maternal immune responses in PTB.

### Hematogenous Dissemination of Inflammatory Products

Clinical attachment loss, as the main periodontal measure, is associated with plasma levels of IL-1β and TNF-α in pregnant women (60), which may promote labor activation through placental and chorion–amnion production of PGE_2_ (61). Women with PTB demonstrated significantly increased GCF levels of IL-6 and PGE_2_ compared with those who had full-term births (9). A systematic review in 2013 (62) reported an association between GCF inflammatory mediator levels and adverse pregnancy outcomes. In a subset of patients with severe periodontitis, locally produced proinflammatory mediators—such as IL-1β, IL-6, and TNF-α—can enter systemic circulation and induce an acute-phase response in the liver that is characterized by an increased level of C-reactive protein (CRP) (63, 64). Serum CRP level was reported to be elevated in subjects with periodontitis (65). An increased CRP level can enhance the risk of cardiovascular disease, cerebrovascular accidents, and PLBW infants (65).

Clinical studies support the association between increased levels of circulating proinflammatory mediators and PTB (66, 67) and have implicated IL-1β and IL-6 as major players in the onset of PTB (68, 69). Moreover, polymorphisms in proinflammatory genes, including the above-mentioned cytokines, have been associated with PTB (70, 71). In addition, elevated amniotic fluid level of IL-6 in the second trimester was associated with the initiation and timing of PTB (72, 73). Therefore, the potential link between periodontitis and PTB may be explained by the following mechanisms. First, periodontal pathogens/byproducts can disseminate toward the placental and fetal tissues. Immune/inflammatory reactions within the placental tissues of the pregnant woman may occur, and the release of proinflammatory mediators in the amniotic fluid may increase and further contribute to PTB. Second, systemic inflammatory changes induced by periodontitis can exacerbate local inflammatory responses within the fetoplacental unit to increase the risk for PTB.

## Experimental Animal Models

The possible roles of periodontitis in PTB have also been explored using experimental animal models. In separate studies, periodontal bacteria were injected into a small chamber in pregnant animals, allowing the establishment of a site of infection distant to the fetal–placental unit to mimic a periodontal infection in a reproducible and simplified manner. These results revealed that maternal infections with periodontal pathogens increase pregnancy complications.

Dental infection of mice with *P. gingivalis* significantly increased levels of circulating IL-1β, IL-6, IL-17, and TNF-α (74). Defects in the placental tissues of *P. gingivalis*-infected mice included degenerative changes in endothelial and trophoblast cells, increased placental detachment, and PROM, and *P. gingivalis* was detected in placental tissues by PCR and immunohistochemistry (74). The *P. gingivalis*-infected group delivered at gestational day (gd) 18.25 versus gd 20.45 for the non-infected control group (*p* < 0.01), with pups exhibiting LBW compared to controls (*p* < 0.01) (74). In another study (75), mice with periodontitis induced by using an inoculum of *P. gingivalis* and *F. nucleatum* exhibited increased circulating levels of IL-6 and IL-8. Similarly, Miyoshi et al. (76) found high levels of contractile-associated proteins and ion channels in the myometrium of PTB model mice with chronic odontogenic *P. gingivalis* infection. In murine models, *F. nucleatum* translocated and caused intrauterine infections (77) and *Campylobacter rectus* significantly decreased fetoplacental weight (78). In a baboon model, a significantly greater frequency of the periodontitis group neonates had decreased gestational age and LBW (79). Spontaneous abortion/stillbirth/fetal demise was increased in the periodontitis group versus the control group (79). Also, combined oral infection of mice with *P. gingivalis* and *C. rectus* significantly reduced overall fecundity compared to controls (80). Overall, most animal studies reported a harmful impact of periodontitis on pregnancy outcome. However, a study performed by Fogacci et al. (81) found no increased risk for PTB or LBW in Wistar rats with induced periodontitis.

## *In Vitro* Models

In addition to the data from animal studies, *in vitro* experiments have been conducted to explore the molecular mechanisms underlying periodontitis-induced PTB. In most *in vitro* models, periodontal pathogens/byproducts were used to infect placental tissues or trophoblast cells/cell lines. These experiments were designed to mimic an *in vitro* periodontal infection in a simplified and reproducible manner to allow investigation of the interaction between periodontal pathogens and placental tissues/cells.

Riewe et al. (82) investigated the transcriptional responses after infection with *P. gingivalis* in extravillous trophoblast (HTR8) cells derived from the human placenta, and found that over 2,000 genes were differentially regulated. *P. gingivalis* induced IL-8 and IFN-γ secretion (83), apoptosis, and arrest in the G(1) phase of the cell cycle (84, 85) in HTR8 cells. Increased IFN-γ secretion and Fas expression occurred in *P. gingivalis*-induced apoptosis of HTR8 cells *via* the ERK1/2 pathway (86). In normal human term fetal membrane explants, *P. gingivalis* may significantly increase TLR7 expression (87).

*Porphyromonas gingivalis* has bioactive components on the cell surface, including lipopolysaccharide, capsules, and fimbriae. *P. gingivalis* lipopolysaccharide induces the production of IL-6 and IL-8 *via* TLR2 in chorion-derived cells (50) and increases expression levels of IL-8, TNF-α, and COX-2 in HTR8 cells in an NF-κB-dependent manner (74). Interestingly, Komine-Aizawa et al. (88) reported that although there is limited direct pathogenic effect of *P. gingivalis* lipopolysaccharide on trophoblast invasion, concurrent smoking reduces trophoblast invasion into the myometrium and thus inhibits maternal vascular reconstruction.

In human placental trophoblast-like BeWo cells, the presence of *A. actinomycetemcomitans* lipopolysaccharide led to increased levels of cytochrome *c*, caspase-2, caspase-3, caspase-9, and BCL2-antagonist/killer 1 mRNA and decreased levels of B-cell CLL/lymphoma 2, BCL2-like 1, and catalase mRNA, consistent with the activation of the mitochondria-dependent apoptotic pathway (89). Additionally, *C. rectus* was reported to effectively invade BeWo cells and upregulate both mRNA and protein levels of IL-6 and TNF-α in a dose-dependent manner (78). Therefore, there is significant evidence that periodontal pathogens and byproducts can induce inflammation and/or apoptosis in placental tissues and cells.

## Effect of Periodontal Treatment on PTB Incidence

Periodontal treatment usually refers to non-surgical periodontal therapy that can improve periodontal health and is defined as plaque removal, plaque control, supragingival and subgingival scaling, root surface debridement, and the adjunctive use of chemical agents. To evaluate periodontitis as a risk factor for PTB, intervention studies were conducted to evaluate the effect of periodontal treatment on the risk of PTB. In these studies, women with preexisting periodontitis were randomly divided into two groups. One received periodontal treatment during or before pregnancy, and the other did not. In this type of study, the researchers could assess if periodontitis was an independent risk factor for PTB by determining if periodontal treatment decreased the incidence of PTB.

In a study published in 2015 (90), the mean gestational age in the periodontal treatment group (treatment performed during the second trimester of the gestational period) was 35.57 ± 2.40 versus 34.17 ± 2.92 weeks in the non-treated group (*p* < 0.05). The treatment group showed a statistically significant reduction in mean CRP levels after delivery compared to baseline values; the control group showed no significant reduction in CRP levels. Another study (91) suggested that periodontal treatment during pregnancy is not only safe for both the child and the mother, but also provides beneficial effects for pregnancy and embryo-fetal development, leading to reduced morbidity and mortality in PTB infants. Macedo et al. (92) also reported an association between a low number of daily tooth brushings and PTB. However, in other recent studies (93–96), periodontal treatment performed on pregnant women was not found to be efficacious in reducing PTB or LBW.

Data from recent systematic reviews are also contradictory. Several reviews (97–99) reported that the risks of perinatal outcomes could be potentially reduced by periodontal treatment in pregnant women, but other reviews (100–103) suggested that periodontal treatment during pregnancy was not an efficient way to reduce the incidence of PTB. However, the evidence was not conclusive due to confounding effects and risks of random errors and bias. Thus, further randomized clinical trials are still necessary.

The preventive effectiveness of periodontal treatment for PTB has still not been established, because it is influenced by many factors such as the diagnostic criteria of periodontitis, microbial community composition, severity of disease, treatment strategy, treatment efficiency, and the timing of treatment during pregnancy (the pre-pregnancy period or during the first or second trimester). Jeffcoat et al. (104) confirmed that decreased PTB may depend on the success of periodontal treatment. In this study of 322 pregnant women with periodontitis, 162 were randomly assigned to receive only oral hygiene instruction and served as the untreated control group, whereas the remaining 160 received scaling and root planing treatment as well as oral hygiene instruction. No significant difference was found between the incidence of PTB in the periodontal treatment group and the control group. However, a logistic regression analysis showed a significant and strong relationship between successful periodontal treatment and full-term birth. Subjects refractory to periodontal treatment were significantly more likely to have PTB. Similarly, in another study (105), periodontal treatment during pregnancy reduced the levels of IL-1β, IL-6, IL-10, and IL-12 in GCF and improved dental parameters. Additionally, the severity of periodontitis was significantly associated with an increased risk of babies born small for gestational age, but no changes in pregnancy-related outcomes were observed following periodontal treatment. These studies suggest the need for the next randomized controlled trials to standardize methodological criteria and utilize a more precise definition of periodontitis. Additionally, for better statistical power, studies should preferably be performed as multicenter studies, and include a large number of participants. Finally, the resulting success or failure of periodontal treatment must be considered.

Preterm birth is the leading cause of infant morbidity and mortality, but classical risk factors explain only one-third of PTB cases, and current intervention strategies have not led to an appreciable reduction of PTB. Therefore, it is necessary to explore mechanisms of causality and generate new hypotheses using an integrated approach. This should be done with increased collaboration among research groups, and using more comprehensive theoretical–methodological approaches to formulate more effective intervention strategies and to detect new risk factors. Although intervention during pregnancy has not consistently been correlated with a reduction in PTB rates, oral health maintenance is an important part of preventive care that is both effective and safe throughout pregnancy and should be supported before and during pregnancy.

## Two New Ideas about the Role of Periodontitis in PTB

### Severe and/or Generalized Periodontitis Promotes PTB

The severity of periodontitis can be categorized based on CAL as follows: mild = 1–2 mm, moderate = 3–4 mm, and severe ≥5 mm (106). CAL is measured with a periodontal probe and is the distance from the base of the probeable crevice to the cementoenamel junction (107). In a case–control study (92), periodontitis that met definition 1 (four or more teeth with at least one site showing CAL of ≥3 mm and probing depth of ≥4 mm) was not associated with fewer weeks of gestation. However, a significant association was found between PTB and periodontitis that was classified according to definition 2 (four or more teeth with at least one site showing CAL of ≥4 mm and probing depth of ≥4 mm). A cohort study (108) of preeclamptic women showed that 49.3% of patients with mild periodontitis and 82.6% of patients with moderate to severe periodontitis delivered preterm. Several studies (47, 109–111) found a highly significant association between PTB and moderate to severe periodontitis. Other studies (112, 113) also suggested that the strength of the association between periodontitis and PTB incidence is higher with increased severity of periodontitis.

Periodontitis can also be defined according to the extent of the disease. Generalized periodontitis is defined as CAL ≥3 mm and probing depth ≥4 mm on four or more teeth and localized periodontitis is defined as CAL ≥3 mm and probing depth ≥4 mm on two or three teeth (6). In a case–control multi-center study (114) of singleton live births, periodontal examinations after delivery identified generalized and localized periodontitis. Generalized periodontitis was identified in 13.4% of PTB women and in 10.8% of control women, and localized periodontitis was identified in 11.6 and 10.8%, respectively (114). A significant association was observed between generalized periodontitis and PTB (114). A case–control study (115) confirmed that only the presence of gingival recession for more than two teeth increased the risk of PTB. In addition, several studies (108, 111, 116) reported greater risk for PTB for mothers if periodontitis progressed during pregnancy.

### Periodontitis Only Promotes PTB for Pregnant Women Who Are Young or HIV-Infected or Have Preeclampsia, Pre-Pregnancy Obesity, or Susceptible Genotypes

Usin et al. (58) reported that the presence of periodontal pathogens in periodontal pockets from pregnant women with different periodontal status was only associated with PLBW infants for young mothers. Pattrapornnan et al. (117) also found a positive risk of PTB, LBW, and PLBW in HIV-infected pregnant women with periodontitis. Nabet et al. (114) demonstrated a significant association between periodontitis and PTB for preeclampsia. Riche et al. (108) and Pattanashetti et al. (118) confirmed that pregnant women with preeclampsia exhibited a greater risk for PTB if periodontitis was present or progressed during pregnancy. Interestingly, Lee et al. (119) reported that pregnant women with periodontitis were 5.56 times more likely to have PTB with preeclampsia than women without periodontitis and that the association was much stronger (odds ratio 15.94) in women with both periodontitis and obesity. In fact, there is a strong association between pre-pregnancy obesity and periodontitis in pregnant females (120).

Genetic factors involved in altered immune response against bacterial infections may also influence the effect of periodontitis in pregnancy. Periodontitis induced by *P. gingivalis* was found to drive periodontal microbiota dysbiosis and cause systemic disease *via* an impaired adaptive immune response in mice (121). Jeffcoat et al. (122) reported a significant relation between a specific polymorphism of prostaglandin E receptor 3 (a gene associated with inflammatory response) and both periodontitis treatment failure and spontaneous PTB.

## Conclusion

Here, for the first time, we describe four possible models of periodontitis in PTB: (1) periodontitis is an independent risk factor for PTB; (2) severe and/or generalized periodontitis promotes PTB; (3) periodontitis only promotes PTB for pregnant women who are young or HIV-infected or have preeclampsia, pre-pregnancy obesity, or susceptible genotypes; and (4) periodontitis has no significant effect on PTB. Because contradictory epidemiological data continue to emerge (model 1 versus 4), future studies should try to test the second and third models, which may, to some extent, explain the conflicting epidemiological data. Although intervention during pregnancy has not consistently been correlated with a reduction in PTB rates, oral health maintenance is an important part of preventive care that is both effective and safe throughout pregnancy and should be supported before and during pregnancy.

## Author Contributions

HR contributed to the literature search, interpretation, writing, and proofreading of the manuscript. MD designed the study and made the ultimate decision on the manuscript.

## Conflict of Interest Statement

The authors declare that the research was conducted in the absence of any commercial or financial relationships that could be construed as a potential conflict of interest.
